# Arterial stiffness in patients with moderate to severe psoriasis on long term treatment with infliximab

**DOI:** 10.1186/s12872-026-06198-6

**Published:** 2026-07-01

**Authors:** Jonar Strand Hagenes, Anja Linde, Kåre Steinar Tveit, Ester Kringeland, Helga Midtbø

**Affiliations:** 1 Department of Clinical Science, University of Bergen, Bergen, Norway; 2 Department of Medicine, Haukeland University Hospital, Bergen, Norway; 3 Department of Dermatology, Haukeland University Hospital, Bergen, Norway; 4 Department of Heart Disease, Haukeland University Hospital, Bergen, Norway

**Keywords:** Psoriasis, Hypertension, Arterial stiffness, Inflammation

## Abstract

**Background:**

Systemic anti-inflammatory therapies reduce arterial stiffness during short term treatment in patients with psoriasis, but less is known about their long-term effects. We assessed arterial stiffness in patients with psoriasis on long-term treatment with infliximab.

**Methods:**

Forty-eight patients with psoriasis (age 46 ± 14 years, 31% women) receiving treatment with infliximab were matched to 103 controls based on age, sex and body mass index (BMI). Arterial stiffness was assessed by carotid-femoral pulse wave velocity (cfPWV). Psoriasis severity was assessed by the psoriasis area and severity index (PASI).

**Results:**

The patients had been treated with infliximab for 4.9 ± 3.9 years and 85% were also treated with methotrexate. Current PASI-score was 0.79 ± 0.75, indicating well treated psoriasis. The prevalence of metabolic syndrome (42%), hypertension (50%) and diabetes (4%) were similar between patients and controls, while a higher proportion of patients were smokers (40% vs. 16%, *p* = 0.003). Average cfPWV was 7.9 ± 1.6 m/s in the psoriasis group and 7.3 ± 1.5 m/s in the control group (*p* = 0.049), both reflecting normal values. In multivariable linear regression analysis in the total study population, having psoriasis remained associated with higher cfPWV (β 0.40, *p* = 0.010) after adjusting for age, sex, systolic blood pressure (SBP), heart rate, BMI and smoking. In patients with psoriasis, higher SBP (β 0.33, *p* = 0.009) and BMI (β 0.27, *p* = 0.021) were associated with higher cfPWV after adjustment for PASI score, age and sex.

**Conclusion:**

In patients with psoriasis on long-term treatment with systemic therapies, arterial stiffness was slightly higher than in matched controls, despite excellent response to treatment and low PASI score.

## Introduction

Psoriasis is a chronic inflammatory disease affecting 2–3% of the population [1]. Patients with psoriasis have a higher prevalence of cardiovascular (CV) disease compared to the general population, partly due to shared risk factors such as obesity and hypertension [2]. Further there is a vast range in disease severity and extent of systemic inflammation. It has previously been shown that having severe psoriasis carries an increased risk of CV mortality [3, 4], while the CV risk in patients with mild psoriasis is less established.

Arterial stiffness is a measure of arterial organ damage and has been shown as a predictor of CV morbidity and mortality [5, 6]. Arterial stiffness has repeatedly been found to be higher in patients with psoriasis compared to controls [7–10]. Disease severity appears to play a role, as higher psoriasis area and severity index (PASI) scores have been associated with greater arterial stiffness [11]. Further, systemic anti-inflammatory therapy seems to impact arterial stiffness positively. A prospective study in 29 patients with psoriasis showed that treatment with biologic therapy, particularly tumor necrosis factor α (TNF-α) inhibitors, led to a reduction in arterial stiffness [12]. This has also been shown in 36 patients with psoriasis arthritis [13]. Further, a recent cross-sectional study in young patients with mild to severe psoriasis reported no difference in arterial function between well-controlled psoriasis patients and healthy controls [14]. However, there is a lack of studies in older patients with more severe psoriasis that are receiving long-term systemic treatment with biologic therapies. Thus, in the present study our aim was to investigate arterial stiffness in patients with moderate to severe psoriasis and well-controlled disease receiving long-term infliximab treatment compared with a matched control group.

## Materials and methods

### Study population

Patients receiving systemic treatment with the Tumor Necrosis Factor α (TNF-α) inhibitor infliximab for moderate or severe psoriasis at the Department of Dermatology, Haukeland University Hospital, Bergen, Norway were invited to participate in the study. Patients with manifest CV disease, defined as: heart failure, previous myocardial infarction or previous cardiac surgery, were excluded. Additional exclusion criteria were current severe psychiatric disorders and expected low compliance. Fifty-five patients were screened for study inclusion, and two patients did not fit the inclusion criteria, resulting in 53 patients with psoriasis included in the study. Psoriasis-free controls for the present study were recruited from the Fat AssociaTed CardiOvasculaR dysfunction study (FATCOR study) at the Department of Heart Disease, Haukeland University Hospital, Bergen, Norway. The FATCOR study has been described previously [15]. In short, FATCOR is an observational, cross-sectional study of 620 women and men without known CV disease aged 30–65 year with a body mass index (BMI) > 27 kg/m^2^. The participants were examined between 2009 and 2016. In total, 106 subjects from FATCOR were matched for sex, age and BMI with the patients with psoriasis. Three control subjects and five patients with psoriasis had missing cfPWV data and were excluded from the analysis. Thus, the final study populations consisted of 48 psoriasis patients and 103 control subjects. The study was approved by the Regional Committee for Medical and Health Research Ethics of Western Norway (REK 2016–02194) and was conducted in accordance with the declaration of Helsinki. All participants signed a written informed consent.

### Cardiovascular risk factor assessment

The participants recorded their medical history on standardized questionnaires that were later quality assured against electronic medical records by the study physician. Height, body weight, waist circumference and clinic blood pressure (BP) were measured by a trained study nurse. BP was measured in accordance with European guidelines on management of hypertension [16]. A clinic systolic BP ≥ 140 mmHg or clinic diastolic BP ≥ 90 mmHg was considered increased. Hypertension was defined as previous history of hypertension, use of anti-hypertensive treatment or increased clinic BP. Diabetes mellitus was considered present if the participant had a known history of diabetes or an elevated blood glycated hemoglobin. Metabolic syndrome was defined according to criteria by the American Heart Association/National Heart, Lung and Blood Institute joint statement [17].

### Assessment of quality of life and severity of psoriasis

Psoriasis severity was assessed by the treating dermatologist in accordance with European guidelines and quantified by PASI [18]. PASI is a validated composite measure of disease severity in psoriasis evaluating percentage of skin involvement, erythema, induration and desquamation, and is currently the most used classification for psoriasis severity. Treatment response was categorized as PASI90, PASI75 and PASI50 defined as a reduction of PASI score by more than 90%, 75% and 50%, respectively [18]. Dermatology Life Quality Index (DLQI) was used to assess quality of life [18].

### Arterial stiffness

CfPWV was measured using a SphygmoCor apparatus (AtCor Medical, Sydney, West Ryde, Australia). Conditions were standardized and speaking or sleeping was not allowed during measurement [19]. Using applanation tonometry, transcutaneous pressure pulse waveforms were recorded from the common carotid artery and the right femoral artery. Simultaneous recording of an electrocardiogram was obtained to synchronize pulse wave times. Curves were optimized by visual inspection of the waveforms following quality control indices. Transit time was measured by the apparatus using the intersecting tangent algorithm. Precise measurements of the distances between the carotid site and the sternal notch and the sternal notch and the femoral site were performed. CfPWV was then calculated as distance in meters between the two recording sites divided by the transit time in seconds [20]. Increased cfPWV was defined as greater than 10 m/s in accordance with guidelines [21]. Augmentation pressure (AP) was calculated as the difference between peak systolic pressure and pressure at the first systolic inflection point. Augmentation index (AIx) was calculated as AP as a percentage of pulse pressure.

### Statistical analysis

Statistical analysis was performed using R v4.5.1 (R Foundation for Statistical Computing, Vienna, Austria) [22]. Continuous data were reported as mean ± standard deviation (SD) for normally distributed variables. Categorical variables were reported as numbers (percentages). Group comparisons were done by the two-sample Student´s *t*-test for continuous variables and Pearson’s chi-square test for categorical variables. For categorical variables with an expected value, in any group, less than five by the Chi-square test, the Fisher exact test was applied. For analysis of covariates of cfPWV in the total study population and in separate analyses in patient group, linear regression models were fitted with standardized data prior to fitting. Selection of variables for the multivariate analysis was based on significance of the t-statistic in univariable analyses, significant difference between groups or relevant clinical variables. Multivariable analyses were run in a correlation matrix with collinearity tools. The variance inflation factors for the individual variables in the multivariable analyses were between 1.1 and 1.5 indicating acceptable levels for collinearity in the models. The data was reported as standardized β-coefficients with 95% confidence intervals (CI). For multivariable models we reported R^2^ and the p-value of the F-test. CfPWV in psoriasis patients and controls were visualised by Sina plots with error bars of the mean with 95% CI, using the ggplot2 and ggforce packages [23, 24]. Differences were considered significant if *p* < 0.05 in all tests.

## Results

### Clinical characteristics and CV risk factors

Patients were on average 46 ± 14 years and 31% were women. On average, psoriasis disease duration was 25 years, ranging from 4 to 52 years. Mean duration of systemic treatment with infliximab was 4.9 ± 3.9 years. 85% of psoriasis patients were also treated with methotrexate. Mean PASI at treatment start was 15 ± 11 and DLQI at treatment start was 17.7 ± 5.9. At the time of cfPWV measurement patients had an average PASI-score of 0.79 ± 0.75 and a DLQI of 0.74 ± 1.41. Thirty-five patients achieved PASI90, indicating excellent treatment response, and only two patients did not achieve a minimum of PASI75. This was also reflected by a low C-reactive protein level that was similar between the patient and the control group (Table 1). A significantly higher proportion of participants in the psoriasis group compared to the control group were current smokers (40% vs. 16%, *p* = 0.003). Otherwise, the prevalence of CV risk factors was similar between groups. The most common CV factors among both psoriasis patients and controls were hypertension (50%) and metabolic syndrome (42%) (Table 1). A similar proportion of hypertension was treated in both groups. There was no difference in classes of antihypertensive treatment used, with angiotensin II receptor blockers most prescribed in both groups.

**Table 1:** Clinical characteristics and measures of arterial stiffness in the study population

	Psoriasis *n* = 48	Control *n* = 103	*p*-value
Demographics			
Age, years	45.6 (14.0)	46.8 (10.6)	0.576
Women, n (%)	15 (31)	31 (30)	1.000
Physical activity, hour/week	9.7 (5.7)	8.8 (5.2)	0.365
Creatinine, µmol/L	74.8 (14.4)	76.3 (12.3)	0.523
LDL mmol/L	3.4 (0.9)	3.6 (0.9)	0.121
HbA_1c_ (%)	5.3 (0.5)	5.5 (0.4)	0.007
C-reactive protein, µg/ml (median, IQR)	1.0 (0.2–2.7)	1.1 (0.45–3.2)	0.192
Duration of psoriasis, years	24.8 (13.0)	-	-
PASI at treatment start	15.3 (10.9)	-	-
Current PASI	0.8 (0.8)	-	-
PASI90, n (%)	35 (73)	-	-
PASI75, n (%)	10 (21)	-	-
PASI50, n (%)	2 (5)	-	-
DLQI at treatment start	17.7 (5.9)	-	-
Current DLQI	0.7 (1.4)	-	-
Psoriasis arthritis, n (%)	21 (44)	-	-
CV risk factors			
Body mass index, kg/m^2^	28.8 (4.8)	29.9 (4.0)	0.153
Smokers, n (%)	19 (40)	16 (16)	0.003
Never smoker (%)	7 (15)	41(43)	0.001
Clinic SBP, mmHg	135(16)	131(17)	0.189
Clinic DBP, mmHg	86 (8)	82 (9)	0.003
Hypertension, n (%)	24 (50)	40 (39)	0.196
Diabetes, n (%)	2 (4.2)	4 (3.9)	1.000
Metabolic syndrome, n (%)	20 (42)	37 (36)	0.498
Medications			
Antihypertensive treatment, n (%)	11 (23)	19 (18)	0.673
Methotrexate, n (%)	41 (85)	-	-
Infliximab, n (%)	48(100)	-	-
Duration of infliximab treatment, years	4.9 (3.9)	-	-
Measures of arterial stiffness			
cfPWV, m/s	7.9 (1.6)	7.3 (1.5)	0.049
Augmentation pressure, mmHg	9 (8)	9 (7)	0.758
Augmentation index, %	23 (15)	24 (16)	0.739
Increased cfPWV, n (%)	3 (6)	4 (4)	0.680

*PASI* Psoriasis Area and Severity Index, *DLQI *Dermatology Life Quality Index, *CV *Cardiovascular, *SBP *Systolic Blood Pressure, *DBP* Diastolic Blood Pressure, *cfPWV* carotid-femoral Pulse Wave Velocity, *IQR* Interquartile range, *LDL* Low density lipoprotein, *HbA*_*1*__c_ Hemoglobin A_1c_

### Prevalence and covariates of arterial stiffness in the total study population

**Fig. 1:**
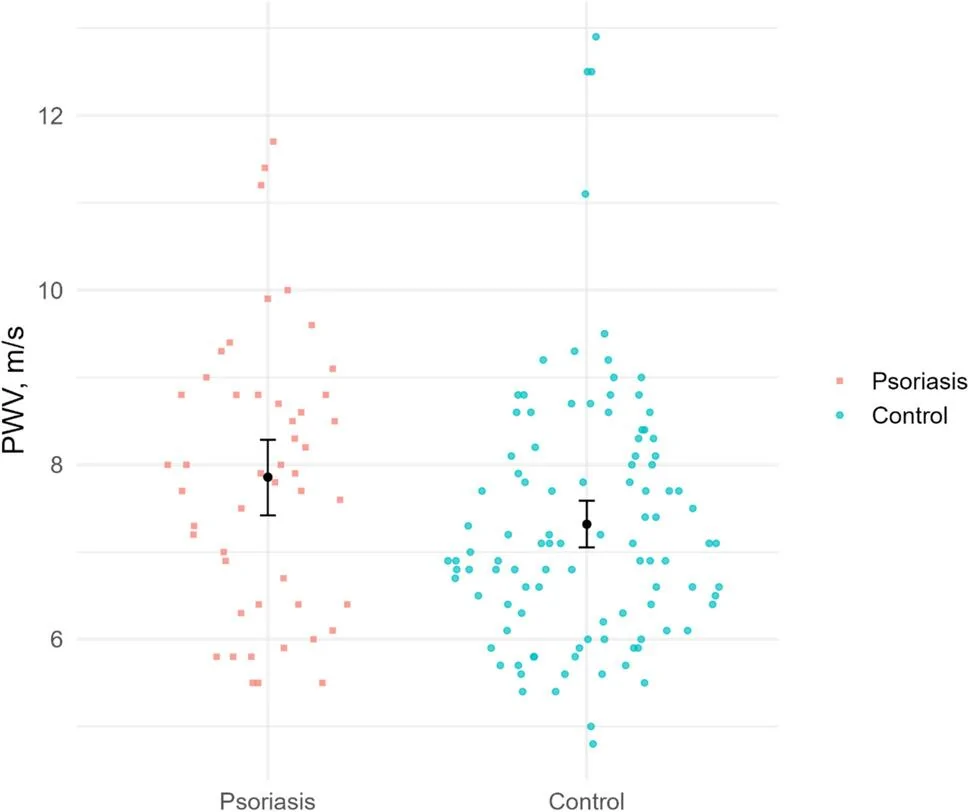
Pulse wave velocity in patients with psoriasis and controls. Sina plot of difference in pulse wave velocity between patients with psoriasis and controls with error bars: Mean ± 95% CI. PWV (Pulse Wave Velocity), CI (Confidence Interval)

Mean cfPWV in the psoriasis group was 7.9 ± 1.6 m/s compared to 7.3 ± 1.5 m/s for controls (*p* = 0.049) (Fig. 1). There was no significant difference in measured AP or AIx between the groups. Very few participants were found to have increased cfPWV; only 3 patients with psoriasis and 4 controls.

**Fig. 2:**
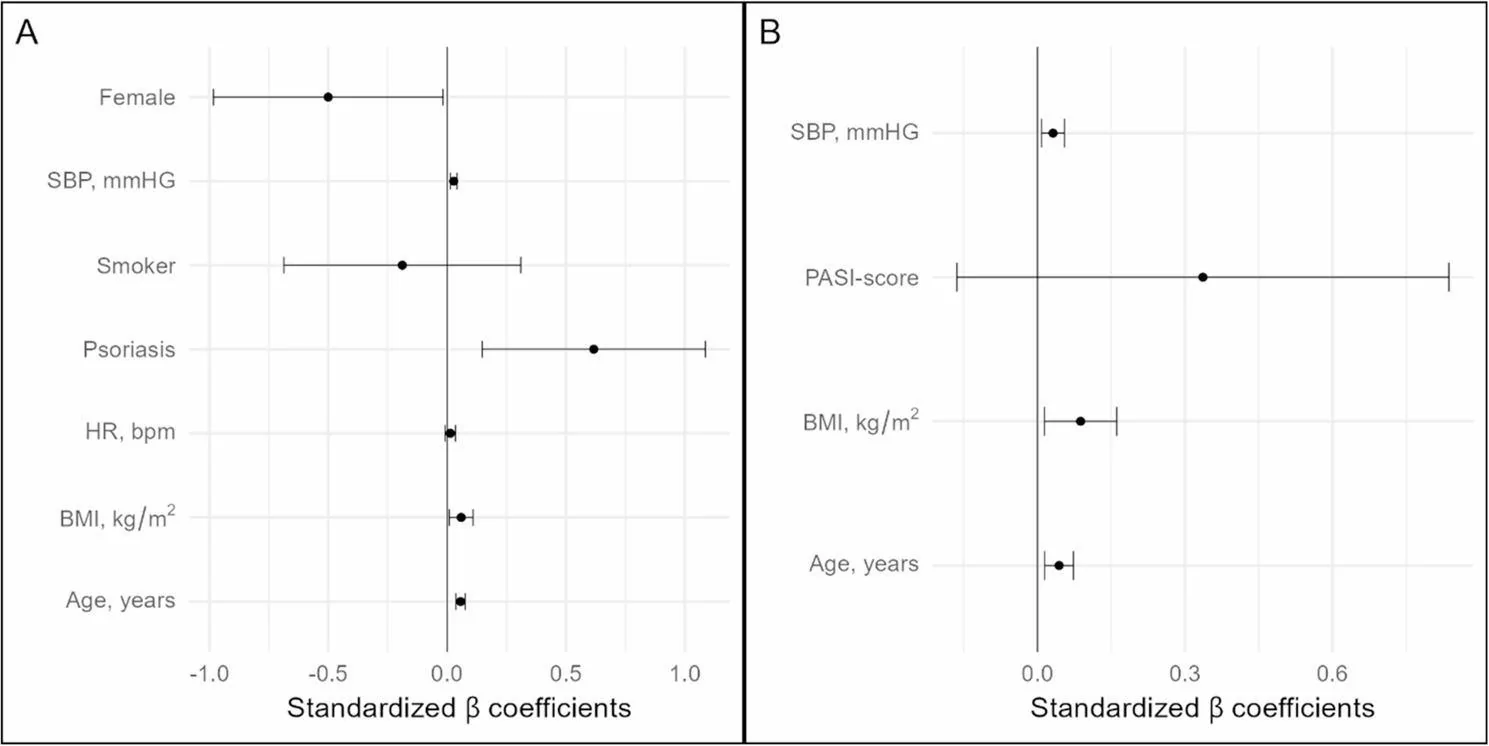
Forrest plot of linear models; linear regression using least squares method on standardized data reported as standardized β and 95% CI. Panel **A** shows the model in the entire study population. Panel **B **shows the model in patients with psoriasis. SBP (Systolic Blood Pressure), PASI (Psoriasis Area and Severity Index), HR (Heart Rate), BMI (Body Mass Index), CI (Confidence Interval)

In univariable linear models in the entire study population, having psoriasis, higher age, heart rate, systolic BP and BMI were significantly associated with cfPWV. In multivariable linear analysis, having psoriasis remained a significantly associated with higher cfPWV (β = 0.40, *p* = 0.010) after adjusting for age, sex, systolic BP, BMI, smoking and heart rate (Table 2) (Fig. 2, Panel A).

**Table 2:** Uni- and multivariable associations of PWV in the total study population

	Univariable		Multivariable	
			Adjusted R^2^: 0.42, *p* < 0.001	
	β (95% CI)	P	β (95% CI)	P
Psoriasis	0.35 (0.01–0.69)	0.044	0.40 (0.10–0.71)	0.010
Age, years	0.49 (0.34–0.63)	< 0.001	0.44 (0.29–0.59)	< 0.001
Women	-0.34 (-0.68–0.01)	0.055	-0.33 (-0.64 – -0.01)	0.042
SBP, mmHg	0.52 (0.38–0.66)	< 0.001	0.30 (0.15–0.45)	< 0.001
BMI, kg/m^2^	0.17 (0.01–0.33)	0.038	0.17 (0.03–0.31)	0.021
Smoker	-0.25 (-0.64–0.14)	0.212	-0.12 (-0.45–0.20)	0.456
Heart rate, beats/minute	0.10 (-0.06–0.27)	0.216	0.09 (-0.05–0.24)	0.212

*SBP* Systolic blood pressure, *BMI* Body mass index, *CI* Confidence Interval

### Prevalence and covariates of arterial stiffness in the psoriasis group

In univariable analysis in the psoriasis group, higher arterial stiffness was significantly associated with higher age, systolic BP and current PASI-score. BMI was borderline significant in association with cfPWV in the univariable analysis (*p* = 0.073), while use of methotrexate or psoriatic arthritis was not. In multivariable analyses in the psoriasis group, PASI-score at the time of PWV-measurement, remained associated with higher cfPWV after adjusting for age, systolic BP and smoking status (β = 0.24, *p* = 0.044). In a secondary multivariable model including BMI instead of smoking status, the association between PASI-score and arterial stiffness was attenuated, while greater BMI emerged as a covariate of cfPWV (β = 0.27, *p* = 0.021) (Table 3) (Fig. 2, Panel B).

**Table 3:** Uni- and multivariable associations of PWV in the patients with psoriasis

	Univariable		Multivariable 1		Multivariable 2	
			Adjusted R^2^: 0.47, *p* < 0.001		Adjusted R^2^: 0.50, *p* < 0.001	
	β (95% CI)	P	β (95% CI)	P	β (95% CI)	P
Age, years	0.56 (0.31–0.81)	< 0.001	0.34 (0.07–0.61)	0.016	0.39 (0.13–0.65)	0.004
SBP, mmHg	0.56 (0.31–0.80)	< 0.001	0.39 (0.14–0.64)	0.003	0.33 (0.09–0.57)	0.009
PASI	0.42 (0.15–0.70)	0.004	0.25 (0.01–0.49)	0.040	0.16 (-0.08–0.40)	0.182
Smoker	-0.37 (-0.98–0.24)	0.229	0.08 (-0.40–0.56)	0.744	-	-
BMI	0.26 (-0.03–0.55)	0.073	-	-	0.27 (0.04–0.49)	0.021
Women	-0.30 (-0.93–0.33)	0.340	-	-	-	-
HR, beats/min	0.09 (-0.21–0.38)	0.564	-	-	-	-

*SBP* Systolic blood pressure, *PASI* Psoriasis Area and Severity Index,*CI* Confidence Interval, *HR* Heart rate

## Discussion

This study found that patients with psoriasis had slightly higher arterial stiffness compared to matched controls despite use of systemic therapies and excellent long-term treatment response. However, only three patients had clinically elevated arterial stiffness. Higher systolic BP, BMI and psoriasis disease severity emerged as the strongest covariables of greater arterial stiffness.

The control subjects in the current study were chosen to match the CV profile of the patients with psoriasis. Due to the cross-sectional design, it is not possible to determine whether the observed differences reflect the cumulative effect of smoking and other CV risk factors, inflammation or psoriasis treatment per se. Of note, very few patients in our study had elevated arterial stiffness indicating generally good arterial health at the individual level despite the observed differences between groups. Although CV risk factors such as smoking status was adjusted for in multivariable analyses, differences in cumulative exposure may contribute to the observed associations, and the potential independent effect of psoriasis on arterial stiffness should therefore be interpreted with caution.

Our finding of slightly higher arterial stiffness in patients with psoriasis is in line with previous cross-sectional studies. In a large substudy of over 2,000 patients with psoriasis from the UK biobank study, arterial stiffness index measured at the index finger was significantly higher in participants with psoriasis than controls [8]. A Turkish study of 50 patients with psoriasis and 50 controls found a significant higher AIx and PWV measured at the brachial artery in patients with psoriasis compared to controls [9]. Additionally, an Italian study of 39 patients with psoriasis compared to 38 controls with a diagnosis of non-psoriasis skin disease found a significant higher cfPWV in patients with psoriasis [10]. Our findings add to these previous publications by demonstrating that arterial stiffness was slightly higher among patients with psoriasis even when on long-term treatment with infliximab and methotrexate. Of note, very few patients in our study had elevated arterial stiffness indicating generally good arterial health at the individual level despite the observed differences between groups.

Contemporary systemic treatment for moderate to severe psoriasis now involves use of even more targeted therapies than TNF-α inhibitors, such as inhibitors of interleukin 17 and 23. The cardiovascular profiles of these drugs are less established than for TNF-α inhibitors and methotrexate, but studies suggest a similar CVD risk profile [25]. Previous studies have demonstrated that systemic therapies could reduce arterial stiffness in patients with psoriasis. A prospective study in 29 patients with severe psoriasis excluding patients with BMI > 35 kg/m^2^ or hypertension, showed that systemic treatment with TNF-α inhibitors decreased arterial stiffness on 6-month follow-up [12]. This has also been shown in 41 patients with inflammatory joint disease, including patients with psoriatic arthritis, treated with infliximab compared to 19 non-randomized controls after 1 year follow-up [13]. However, few studies have compared patients with well-controlled psoriasis with healthy controls. A recent paper by Merzel Šabović et al. found no difference in arterial stiffness between young, healthy patients with psoriasis with minimal disease activity and healthy controls, although there were power issues due to the design of the study [14]. Our study, including patients with comorbid traditional CV risk factors in a larger study population, contrast these findings and add to the literature by showing that even among patients with long-term, well-controlled psoriasis receiving systemic therapies, arterial stiffness was slightly higher than in matched controls. The control subjects in the current study were chosen to match the CV profile of the patients with psoriasis. Due to the cross-sectional design, it is not possible to determine whether the observed differences reflect the cumulative effect of smoking and other CV risk factors, inflammation or psoriasis treatment per se.

Higher BMI was a major covariate of arterial stiffness in both patients and controls in the present study. Only nine pariticipants were normal weight in the study, limiting the generalizability of our findings to a healthier population and potentially attenuating the differences in arterial stiffness between groups. These points should be considered when interpreting the relatively modest effect sizes observed between groups. Higher BMI has previously been linked to greater arterial stiffness in large prospective analyses. However, this effect was modulated predominantly through elevated BP [26]. In the present study, systolic BP was also significantly associated with arterial stiffness however, the association between higher BMI and cfPWV was independent of BP. Obesity is associated with low-grade inflammation [27]. In patients with psoriasis, obesity has been linked both to the incidence and severity of psoriasis [2]. Obesity and higher BMI have also been linked to greater risk of treatment discontinuation and lower treatment response with infliximab treatment [28, 29]. In our study, the association between PASI and higher arterial stiffness was attenuated when adjusting for BMI, this could suggest a potentially mediating role of adiposity. Consequently, the independent contribution of psoriasis-related inflammation to arterial stiffness should be interpreted with caution. This also raises questions whether the low-grade inflammation associated with increased body weight modulates both psoriasis disease activity, treatment response and arterial damage. Supporting this, weight loss has been demonstrated to lower disease severity and increasing quality of life in psoriasis [30]. Further, weight loss has also been proposed as a possible intervention on arterial stiffness [31].

### Strengths and limitations

This study is of a cross-sectional design and causality cannot be established. Despite careful matching between the patients and controls and multivariable adjustments, residual confounding may remain, particularly related to cumulative smoking exposure, metabolic risk factors such as duration of hypertension and cumulative inflammatory burden. The cohort consisted of all patients at Haukeland University Hospital, Bergen Norway that received systemic therapy with infliximab that were without manifest CV disease. A very high proportion of patients were also treated with methotrexate, and this study cannot distinguish effects of methotrexate and effects of infliximab, The current results might not apply to less selected patients with psoriasis encountered in clinical practice. Haukeland is a tertiary hospital in the Western health region of Norway serving a population of 655 thousand people, but a multicenter study could have increased the size of the study cohort and the power of the study. The current study does have limited statistical power for sensitivity analyses or relevant subgroups analyses. Type 2 errors could not be ruled out. The study population consisted predominantly of males, reflecting that more males than females receive systemic treatment for psoriasis [32, 33]; this limited our ability to study potential sex differences.

Strengths of the study include the use of gold standard cfPWV to measure aortic arterial stiffness [19]. The control group was well matched to the psoriasis group regarding prevalence of CV risk factors, ensuring comparability between groups.

## Conclusion

Patients with psoriasis had slightly higher arterial stiffness compared to matched controls despite long-term therapy with infliximab and excellent treatment response. However, the prevalence of increased arterial stiffness was low among patients, suggesting that the difference between groups likely reflects subtle vascular alterations rather than overt arterial organ damage. Higher systolic BP, BMI and psoriasis severity emerged as the strongest covariables of greater arterial stiffness, emphasizing the importance of managing modifiable risk factors to optimize CV health in patients with psoriasis.

## Data Availability

The datasets generated and/or analysed during the current study are not publicly available because the Regional Committee for Medical and Health Research Ethics of Western Norway does not allow for public sharing of the data. The data are available from the corresponding author upon reasonable request.

## Abbreviations

BMI: Body mass index
PASI: Psoriasis area and severity index
cfPWV: Carotid–femoral Pulse Wave Velocity
BP: Blood pressure
SBP: Systolic blood pressure
CV: Cardiovascular
TNFα: Tumor necrosis factor alpha
FATCOR-study: Fat Associated Cardiovascular Risk–study
REK: Regional committees for medical and health research ethics
DLQI: Dermatology life quality index
AP: Augmentation pressure
AIx: Augmentation index
SD: Standard deviation
CI: Confidence interval
PWV: Pulse wave velocity
HR: Heart rate
RN: Registered nurse
MD: Medical doctor
DBP: Diastolic blood pressure
